# ZFP36L1 Negatively Regulates Plasmacytoid Differentiation of BCL1 Cells by Targeting BLIMP1 mRNA

**DOI:** 10.1371/journal.pone.0052187

**Published:** 2012-12-20

**Authors:** Asghar Nasir, John D. Norton, Maria Baou, Anna Zekavati, Marie-Jose Bijlmakers, Steve Thompson, John J. Murphy

**Affiliations:** 1 Division of Immunology, Infection and Inflammatory Disease, King's College London, London, United Kingdom; 2 Department of Biological Sciences, University of Essex, Colchester, Essex, United Kingdom; 3 Department of Biomedical Sciences, University of Westminster, London, United Kingdom; University of Hawaii Cancer Center, United States of America

## Abstract

The ZFP36/Tis11 family of zinc-finger proteins regulate cellular processes by binding to adenine uridine rich elements in the 3′ untranslated regions of various mRNAs and promoting their degradation. We show here that ZFP36L1 expression is largely extinguished during the transition from B cells to plasma cells, in a reciprocal pattern to that of ZFP36 and the plasma cell transcription factor, BLIMP1. Enforced expression of ZFP36L1 in the mouse BCL1 cell line blocked cytokine-induced differentiation while shRNA-mediated knock-down enhanced differentiation. Reconstruction of regulatory networks from microarray gene expression data using the ARACNe algorithm identified candidate mRNA targets for ZFP36L1 including BLIMP1. Genes that displayed down-regulation in plasma cells were significantly over-represented (P = <0.0001) in a set of previously validated ZFP36 targets suggesting that ZFP36L1 and ZFP36 target distinct sets of mRNAs during plasmacytoid differentiation. ShRNA-mediated knock-down of ZFP36L1 in BCL1 cells led to an increase in levels of BLIMP1 mRNA and protein, but not for mRNAs of other transcription factors that regulate plasmacytoid differentiation (xbp1, irf4, bcl6). Finally, ZFP36L1 significantly reduced the activity of a BLIMP1 3′ untranslated region-driven luciferase reporter. Taken together, these findings suggest that ZFP36L1 negatively regulates plasmacytoid differentiation, at least in part, by targeting the expression of BLIMP1.

## Introduction

The ZFP36/Tis11 zinc finger protein family bind to adenine uridine (AU)-rich elements (AREs) in the 3′ untranslated regions of mRNAs and mediate ARE-mediated mRNA decay [1]. There are four mammalian members of the ZFP36 family that include the prototype, ZFP36 (Tis11, TTP, Nup475, GOS24), ZFP36L1 (Tis11b, BERG36, ERF-1, BRF-1) and ZFP36L2 (Tis11d, ERF-2, BRF-2). The fourth family member described in rodents, ZFP36L3, is expressed in mouse placenta, but apparently not in human placenta or other human tissues [2].

Binding of ZFP36/Tis11 proteins to AREs of their target mRNAs promotes deadenylation, decapping and finally, degradation by either exosome (3′-5′ degradation) or XRN1 exonuclease (5′-3′ degradation) [3]–[5]. In addition, some ZFP36/Tis11 family members attenuate translation of their target mRNAs by inhibiting recruitment to polyribosomes [6]. The post-transcriptional regulatory functions of ZFP36/Tis11 proteins have also been reported to overlap and interact with those of microRNAs [7]. Sets of mRNA targets for individual ZFP36/Tis11 family members have been identified in several studies [8], [9] are reviewed in [4], [5] and include a number of key cytokines such as IL-2 [10], IL-3 [11], [12], IL-10 [9]. An emerging picture is that each ZFP36/Tis11 protein targets a distinct, but overlapping repertoire of probably several hundred mRNAs. Cell-type-specificity is further imparted by the distinct expression pattern of each family member.

We originally identified the human *ZFP36L1* gene (*10A/Berg36*) from a panel of cDNAs representing early response genes from activated B lymphocytes [13], [14]. ZFP36L1 is essential for embryonic development [6], [15] and in common with other ZFP36/Tis11 family members, possesses proapoptotic functions [14], [16], [17]. In B cells, expression of ZFP36L1 is inducible by a diverse range of activating signals [13], [14], [17], [18], suggesting that it may serve additional biological roles in this cell lineage. Prompted by recent data showing that ZFP36L1 serves an essential role in maintaining the undifferentiated state of myeloid [19] and pluripotent embryonic stem cells [20], we have investigated a possible role for this protein in B cell plasmacytoid differentiation using the mouse BCL1 cell line. These cells can be induced to undergo plasmacytoid differentiation in response to cytokines (IL-2/IL-5) and are a useful *in vitro* model system to study plasma cell differentiation [21]. We report here that ZFP36L1 negatively regulates plasmacytoid differentiation, at least in part by targeting mRNA for the plasma cell regulatory transcription factor, BLIMP1.

## Materials and Methods

### Cell Culture

BCL1 mouse B cell leukemia cells were obtained from the European collection of cell cultures (ref. No. 90061904) and maintained in RPMI 1640, 10% FBS, 50 U/ml penicillin/streptomycin, 2 mM LGlutamine,1% sodium pyruvate, 1% non-essential amino acids, and 0.05 mM 2-mercaptoethonal. Ramos cells (European collection of cell cultures ref. No. 85030802), and SEM [22], Nalm6 [23], JJN3 [24], KMM1 [25], MM1S (ATCC, CRL-2974), RPMI-8226 (ATCC, CCL-155) and KMS-11 [26] cells (all obtained from Prof. K Yong, Dept. of Haematology, University College London, UK) were maintained in RPMI 1640, 10% FBS, 50 U/ml penicillin/streptomycin and 2 mM L-Glutamine. B Cells were isolated from spleens of C57BL/6 mice using DynalR Mouse B-Cell Negative Isolation Kit (Invitrogen, Paisley, UK). Primary splenic murine B Cells were maintained in RPMI 1640, 10% FBS, 50 U/ml penicillin/streptomycin and 0.05 mM β-mercaptoethanol. BCL1 cells seeded at 2×10^5^ cells/ml were stimulated with 20 ng/ml recombinant mouse Interleukin-2 (R&D Systems, Abingdon, UK) and 5 ng/ml recombinant mouse Interleukin-5 (R&D Systems). Primary murine splenic B cells seeded at 1×10^6^ cells/ml were stimulated with 10 µg/ml lipopolysaccharide (Sigma-Aldrich, Poole, UK).

### Over-expression of ZFP36L1 in BCL1 cells

A cDNA encoding the ZFP36L1 open reading frame was cloned into the pcDNA3 plasmid (pcDNA3ZFP36L1) and this or empty pcDNA3 was co-transfected with pcDNA3EGFP plasmid into BCL1 cells by electroporation. After 24h, transfected cells were sorted on the basis of EGFP expression using a flow Dako Cytomation Mo-Flo Fluorescence Activated Cell Sorter. Sorted BCL1 transfected cells were cultured in medium alone or with IL-2 and IL-5 for up to four days.

### Modulation of ZFP36L1 Levels using a shRNA Expressing Lentivirus in BCL1 cells

zfp36l1-shRNA lentiviruses were constructed by cloning zfp36l1 shRNAs (Table S1) into the pSicoR lentiviral vector [27]. 10 µg lentiviral plasmid DNA pSicoR and 5 µg each of the packaging plasmid DNA (pMDLg/pRRE, pRSV-Rev and pMD2.G) were added to HEK 293T cells for production of virus as described previously [27]. Stable mammalian BCL1 cell lines containing the zfp36l1-shRNAs, empty lentivirus or a scrambled version of the zfp36l1-shRNA were prepared following transduction of cells with virus and cell sorting GFP positive cells on a flow cytometer. Efficiency of ZFP36L1 knockdown was assessed by qRT-PCR and Western Blot analysis.

### Western Blot Analysis

Western Blot analysis was carried out as previously described [14]. Protein extracts were separated by SDS-PAGE Electrophoresis and electrotransferred onto nitrocellulose membranes (ProtranR, Schleicher and Schuell, Dassel, Germany) which were blocked overnight at 4°C in blocking buffer containing 5% milk powder and TTBS buffer (23 mM Tris base, 0.5 M NaCl, and 0.05% Tween-20). Nitrocellulose membranes were incubated with appropriate dilution of the following primary antibodies for 1h; rabbit polyclonal anti-BRF1/2 (ZFP36L1/2) (Cell Signalling, Danvers, MA, USA), goat anti-ZFP36 (Santa Cruz Biotechnology, Santa Cruz, CA, USA), rabbit anti-BLIMP1 (Cell Signalling). Rabbit anti-HSP90 (Santa Cruz Biotechnology) and rabbit anti-human βACTIN antibodies (Cell Signalling) were used as loading control antibodies. Following this incubation, membranes were washed and incubated with horseradish peroxidise-conjugated swine anti-rabbit or donkey anti-goat immunoglobulin for 1 h. Chemiluminescent signals on membranes were detected using SuperSignalR West Femto Substrate (Pierce, Rockford, IL,USA) and exposed to X-Ray films (Kodak Biomax MS, Sigma). The films were developed in a Xograph Compact x4 film processor (Xograph Imaging Systems Ltd. Tetbury, UK).

### Enzyme Linked Immunosorbent Assay (ELISA) for Measurement of Secreted IgM

ELISA was performed in 96-well Maxisorb Immunoplates (Nunc. Ltd, Bedford, UK). The plates were coated with rat anti-mouse IgM antibody (BD Biosciences) and kept overnight at 4°C in a damp box. Wells were then washed three times with PBS/0.1% Tween20, and were then blocked with PBS/1% BSA for 1 h at 37°C. After washing the plates four times with PBS/0.1% Tween20 different concentration of mouse IgM (Sigma-Aldrich) or cell supernatant samples were added to wells and the plates incubated at 37°C for 1 h. Plates were then washed four times with PBS/0.01% Tween20 and 100 µl of goat anti-mouse IgM Peroxidase Conjugate antibody (Sigma-Aldrich) was added to each well. The plates were then incubated at 37°C for 1 h and 30 min. Plates were washed five times with PBS/0.1% Tween20, and 50 µl of 3,3′,5,5′ Tetramethylbenzidine (TMB) was added to each well. The reaction was terminated with 50 µl/well of 2 M sulphuric acid and the optical densities read at 450 nm using a Multiscan plate reader. Concentrations of IgM in cell supernatants were calculated by reference to the standard curve.

### RT-PCR Analysis

Total RNA was extracted from cells using an RNeasy Mini Kit (Qiagen, Crawley, UK). 1 µg of total RNA was reverse-transcribed for cDNA synthesis using a QuantiTect Reverse Transcription kit (Qiagen). PCR was carried out in a Biometra UNO II thermocycler (Biometra, Gottingen, Germany) in a total volume of 20 µl containing; 2 µl Reaction buffer (l00 mM Tris-HCl, 500 mM KCL, 15 mM MgCl2, 1% Triton X-100), 800 µM PCR nucleotide mix (final concentration of 200 µM of each dNTP), 0.5 µM of each primer, 50–200 ng of template DNA, 2.5 U Taq DNA polymerase, and nuclease-free water. The thermal cycling profile was 1 cycle of 95°C for 2 min, followed by 30 cycles of 94°C for 30 sec, 56°C for 30 sec, and 72°C for 1 min. The primers used were; human ZFP36L1 primers, forward 5′- GATGACCACCACCCTCGT-3′ and reverse 5′ CTGGGAGCACTATAGTTGAGCA-3′, human BLIMP1 primers, forward 5′-ACGTGTGGGTACGACCTTG-3′ and reverse 5′-CTGCCAATCCCTGAAACCT-3′ human β ACTIN primers, forward 5′-CCAACCGCGAGAAGATGA-3′ and reverse 5′-CCAGAGGCGTACAGGGATAG-3′.

### Real-time RT-PCR Analysis

Wild-type, control empty lentivirus, scramble lentivirus and zfp36l1 shRNA knockdown BCL1 were treated with and without cytokines for various time periods. 1 µg of total RNA was reverse transcribed for cDNA synthesis using a QuantiTect Reverse Transcription kit (Qiagen). Real-time quantitative RT-PCR (qRT-PCR) was performed using an ABI PrismR 7000 Sequence Detection System (Applied Biosystems, Carlsbad, CA, USA), in a total volume of 25 µl containing 500 ng of cDNA, 2× QuantiTectR SYBRR Green Master mix (Qiagen), and 0.5–1 µM of each primer. All PCR reactions were carried out in triplicate. The PCR program consisted of 1 initial cycle of 15 min at 95°C, 40 cycles of 15 sec at 95°C, 15 sec at 60°C, and 30 sec at 72°C. The relative changes in gene expression levels were calculated using the 2^–ΔΔCT^ method [28]. The primer pairs used for qRT-PCR were as follows; mouse zfp36l1 forward 5′-TTCACGACACACCAGATCCT-3′ and reverse 5′-TGAGCATCTTGTTACCCTTGC-3′, mouse blimp1 5′-GGCTCCACTACCCTTATCCTG-3′ and reverse 5′-GTTGCTTTCCGTTTGTGTGA-3′, mouse xbp1 forward 5′-TGACGAGGTTTCAGAGGTG-3′ and reverse 5′-TGCAGAGGTGCACATAGTCTG-3′, mouse irf4 forward 5′-ACAGCACCTTATGGCTCTCTG-3′ and reverse 5′-ATGGGGTGGCATCATGTAGT-3′, bcl6 primers, forward 5′CTGCAGATGGAGCATGTTGT 3′and reverse 5′GCCATTTCTGCTTCACTGG3′ and mouse β actin primers, forward 5′-CTAAGGCCAACCGTGAAAAG-3′ and reverse 5′-ACCAGAGGCATACAGGGACA-3′.

### 3′UTR Luciferase Reporter Assay

The human BLIMP1 3′UTR was cloned into the pMIR luciferase reporter vector to generate pMIRBLIMP1 3′UTR luciferase reporter vector (cat. No. SC18855, OriGene Technologies Inc, Rockville, USA). 24 h prior to transfection HEK 293T cells (2×10^5^ cells/ml) were seeded per well of a 12 well plate. Cells were then transfected with 100 ng of pMIRBLIMP1 3′UTR luciferase reporter vector alone or with 200 ng of human ZFP36L1 expression vector (pcDNA6ZFP36L1) or ZFP36L1 with a zinc finger domain mutation (pcDNA6ZFP36L1 Mut). A renilla expression vector (10 ng, pRL-CMV, Promega,) was also included in all transfections. Cells were left for 24 h after transfection, then cell lysates were prepared, and luciferase and renilla signals measured using the Dual luciferase reporter assay syatem (Promega) on a Fluostar Optima (BMG, Labtech) plate reader.

### Reverse Engineering of a ZFP36L1 Gene Regulatory Network in B cells

A U133A (Affymetrix, Santa Clara, CA, USA) expression profiling microarray dataset (GSE6691) of 56 samples representing primary normal and malignant mature B and plasma cells [29] was used for analysis. Pre-processing by filtering of RMA-normalised log-transformed data for ‘present’ calls and subsequent analysis was performed using the GenePattern suite of software tools [30]. Differentially expressed genes were identified by using the ComparativeMarkerSelection module [31] using a T-test statistic with a Bonferroni-corrected P value cut-off of 0.05 or a false discovery rate (FDR) cut-off of 0.01. The microarray dataset was digitally deconvoluted using the ARACNe algorithm [32], [33] with a mutual information (MI) threshold of 0.41 and a data processing inequality (DPI) tolerance of 0.175 together with an expanded list of 1300 human transcription factors for DPI correction [34]. The resulting network was visualised and further analysed using the Cytoscape (version 2.6.0) graph mapping platform [35]. Enrichment of Gene Ontology terms for ARACNe-inferred ZFP36L1 targets was determined using the GO-TermFinder algorithm [36].

### Statistical Analysis

A students T test was performed wherever applicable. The *p* values are indicated as follows: *p<0.05, **p<0.01, ***p<0.001.

## Results

### ZFP36L1 Expression During B cell Differentiation

**Figure 1:**
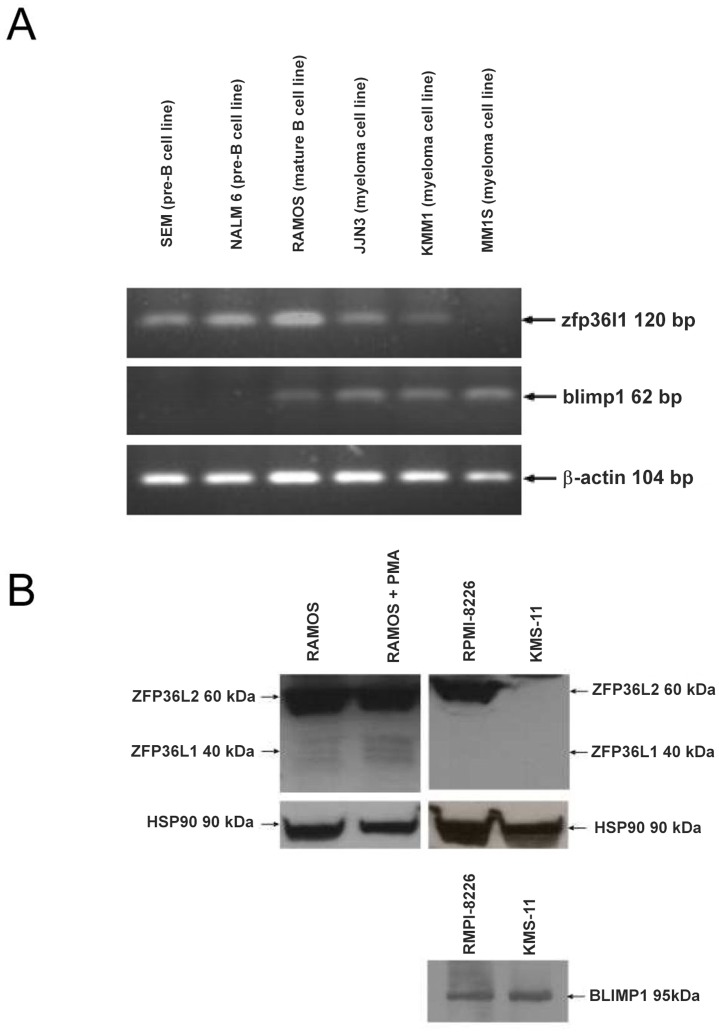
ZFP36L1 expression in human B cells at various stages of differentiation. (A) RT-PCR analysis of ZFP36L1 expression in human malignant B cells representing different stages of B cell differentiation. For comparison BLIMP1 expression was also measured. β ACTIN expression levels are shown as a loading control. (B) Comparison of ZFP36L1 protein levels in human Ramos B cells before and after 3 h PMA stimulation and in two myeloma cell lines RPMI-8226 cells and KMS-11. BLIMP1 expression is also shown in RPMI-8226 cells and KMS-11 cells. HSP90 levels are shown as a loading control. ZFP36L1/L2, HSP90 and BLIMP1 proteins were detected by anti-BRF1/2, anti-HSP90 and anti-BLIMP1 antibodies respectively. Anti-BRF1/2 antibody cross-reacts with ZFP36L2 (approx. 60 kDa) and ZFP36L1 appears typically as a constellation of induced bands (approx. 40 kDa) in human cells.

**Figure 2:**
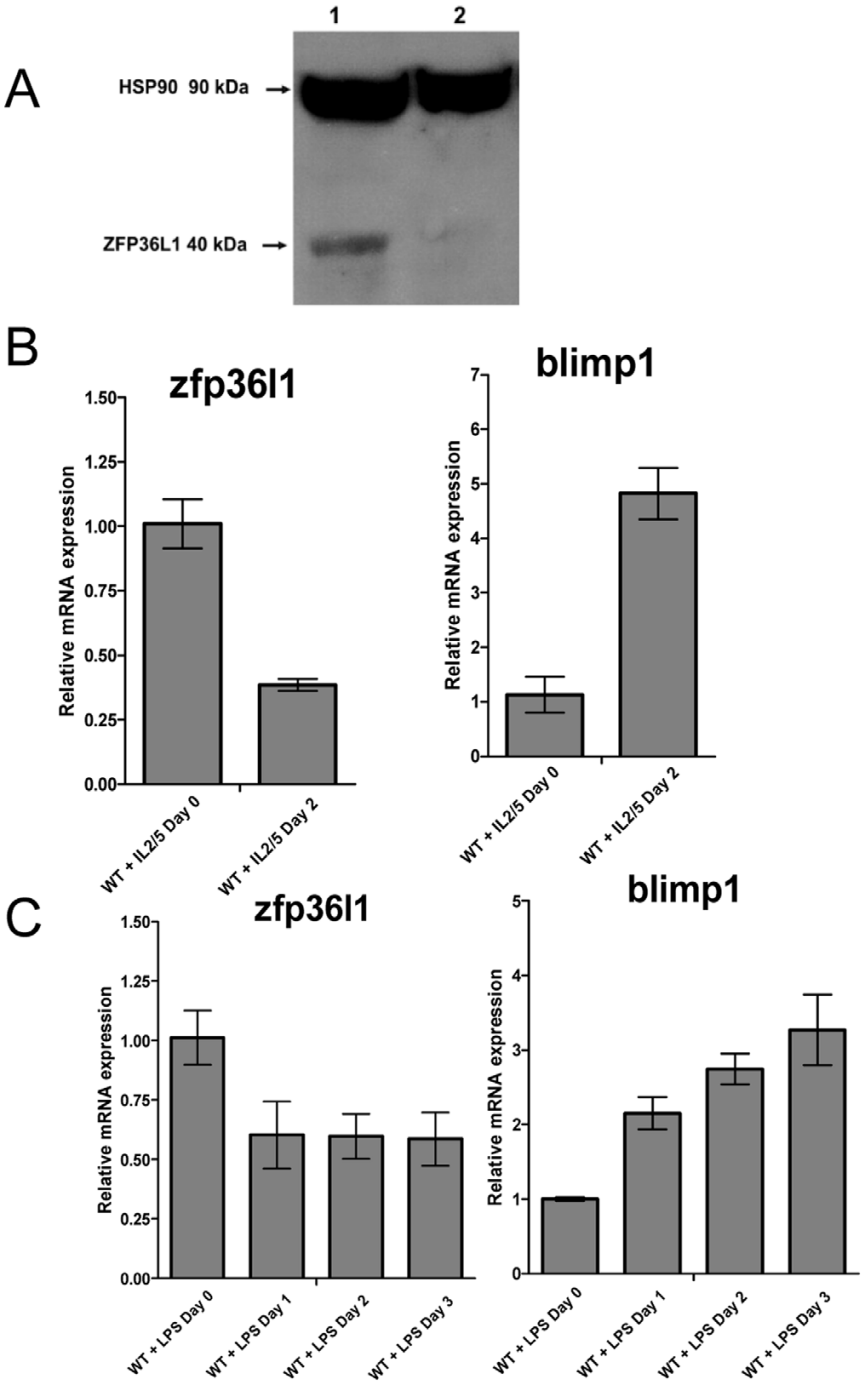
Significant **downregulation of ZFP36L1 is associated with plasmacytoid differentiation of B cells.** (A) Western Blot analysis of ZFP36L1 expression in IL-2/5 treated murine leukemic BCL1 cells. Protein lysates were made from unstimulated cells (lane 1) or from cells 96 h after stimulation with cytokines (20 ng/ml IL-2 and 5 ng/ml IL-5) (lane 2). ZFP36L1 and HSP90 proteins were detected by anti-BRF1/2 and anti-HSP 90 antibodies respectively. (B) qRT-PCR analysis of zfp36l1 and blimp1 mRNA expression in day 0 versus 48 h IL-2/5 stimulated BCL1 cells. (C) qRT-PCR analysis of time course of zfp36l1 and blimp1 mRNA expression over 3 days in LPS (10 µg/ml) stimulated murine splenic B cells. The 2^–ΔΔCT^ method of relative quantification was used to determine the fold change in mRNA expression. The results shown were normalized to β-actin mRNA expression. The results show mean ±SD from one representative experiment.

The transcription factor, BLIMP1, drives differentiation of B cell to plasma cells [37]–[39]. We therefore initially evaluated expression of ZFP36L1 in parallel with BLIMP1 at different stages of B cell differentiation by RT-PCR using human B cell tumour cell lines representing stages of B cell development from pre-B to plasma cells. As shown in Fig. 1A, ZFP36L1 mRNA was detected at high levels in the pre-B cell lines, SEM and NALM 6 in which expression of BLIMP1 mRNA was absent. RAMOS mature B cells expressed the highest level of ZFP36L1 with detectable expression of BLIMP1 mRNA. All three cell lines of myeloma origin (representing plasma cells) expressed higher levels of BLIMP1 with either lower (JJN3, KMM1) or absent (MMIS) expression of ZFP36L1 mRNA. Thus, although the expression patterns of ZFP36L1 and BLIMP1 were not mutually exclusive, there appeared to be an overall trend of ZFP36L1 expression that was reciprocal to that of the plasma cell regulator, BLIMP1 and that decreased in cell lines of plasma cell phenotype. Analysis of ZFP36L1 protein also revealed very low/absent levels of ZFP36L1 in myeloma cells compared to unstimulated and PMA-stimulated Ramos Burkitt lymphoma cells (Fig. 1B). We next evaluated whether induced plasmacytoid differentiation of the murine BCL1 cell line is accompanied by down-regulation of ZFP36L1. BCL1 cells expressed relatively high levels of zfp36l1 mRNA and protein that were both down-regulated following IL-2/5 stimulation when blimp1 mRNA expression was upregulated (Fig. 2A and B). With regard to the two other members of the ZFP36 family, ZFP36L2 was undetectable in BCL1 cells (Fig. 2A) whereas ZFP36 was expressed but levels of expression were unaffected by IL-2/5 stimulation (Fig. S1). Stimulation of primary murine B lymphocytes with LPS also resulted in a reduction in zfp36l1 mRNA that was essentially complete by day 1 post-stimulation. Blimp1 expression was up-regulated (2.5-fold) at this time point and continued to accumulate over the following 2 days (Fig. 2C). Finally, immunohistochemical analysis revealed high levels of ZFP36L1 protein expression in human tonsil sections that was predominantly localised to proliferating B cells in germinal centres (Fig. S1).

### ZFP36L1 Regulates the Differentiation of BCL1 cells

**Figure 3:**
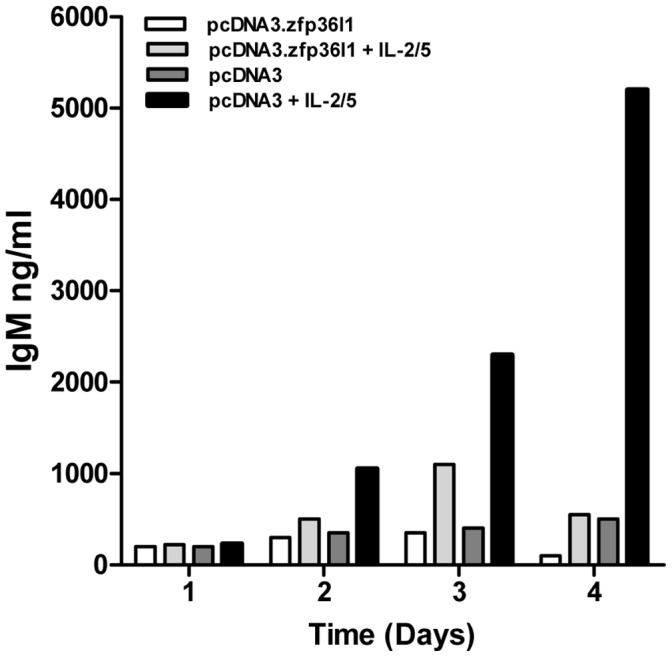
Ectopic expression of ZFP36L1 in BCL1 cells inhibits cytokine-induced. IgM production. Levels of IgM production in ZFP36L1 transfected BCL1 stimulated with IL-2/5 (20 ng/ml IL-2 and 5 ng/ml IL-5) over 4 days compared to levels produced by empty vector BCL1 cells cultured under the same conditions. Cells were co-transfected with either pcDNA3.ZFP36L1 or empty pcDNA3 vector and pcDNA3.EGFP and then sorted on the basis of EGFP expression before been set up in culture in the absence or presence of cytokines. Cells were cultured at 4.0×10^4^/ml and ELISA measurements were made in duplicate. The results shown are representative of 3 similar experiments.

To determine whether down-regulation of ZFP36L1 is required for B cell differentiation, we evaluated the effect of transfecting BCL1 cells with the vector, pcDNA3ZFP36L1, encoding exogenous ZFP36L1 in combination with an EGFP expressing vector. Following culture of EGFP positive cell sorted populations in the presence of IL-2/5, the extent of IgM production at days 3–4 was markedly reduced in pcDNA3ZFP36L1-expressing cells compared with control cells expressing empty vector, pcDNA3 (Fig. 3). Ectopic expression of ZFP36L1 did not affect cell viability or proliferation of BCL1 cells under these conditions (data not shown). The impaired differentiation of pcDNA3ZFP36L1-expressing cells implies that down-regulation of ZFP36L1 is normally required for plasmacytoid differentiation; the higher levels of ZFP36L1 seen in BCL1 cells prior to differentiation induction implicates ZFP36L1 function in maintaining the immature differentiation state of BCL1 cells.

**Figure 4:**
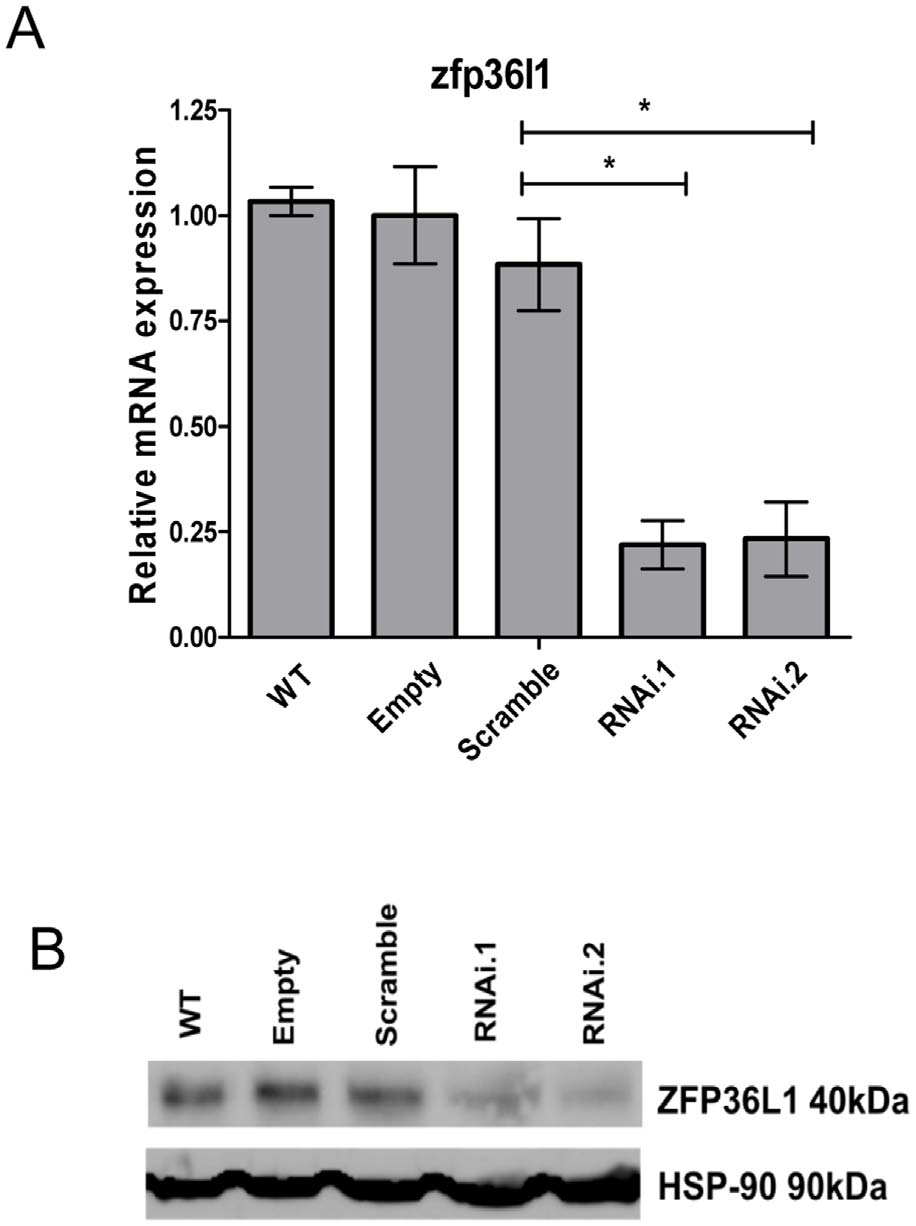
Effective downregulation of zfp36l1 mRNA and protein expression by zfp36l1 shRNA lentivirus (pSicoR.zfp36l1) in independent lentiviral transduced BCL1 cell lines. (A) qRT-PCR analysis of zfp36l1 mRNA levels in pSicoR.zfp36l1.RNAi1 and pSicoR.zfp36l1.RNAi2 lentivirus infected cells compared to wild-type, empty vector or pSicoR.scramble.RNAi infected cells. The results represent mean ±SD (n = 3) of zfp36l1 mRNA levels in three independent cells lines generated by three independent rounds of lentiviral infection for each cell type (apart from wild-type). * = p<0.05 as determined by t-test. (B) Western blot analysis of ZFP36L1 protein expression in wild-type, empty vector, scramble, pSicoR.scramble.RNAi1and pSicoR.scramble.RNAi2 cells. HSP90 levels are shown as a loading control.

**Figure 5:**
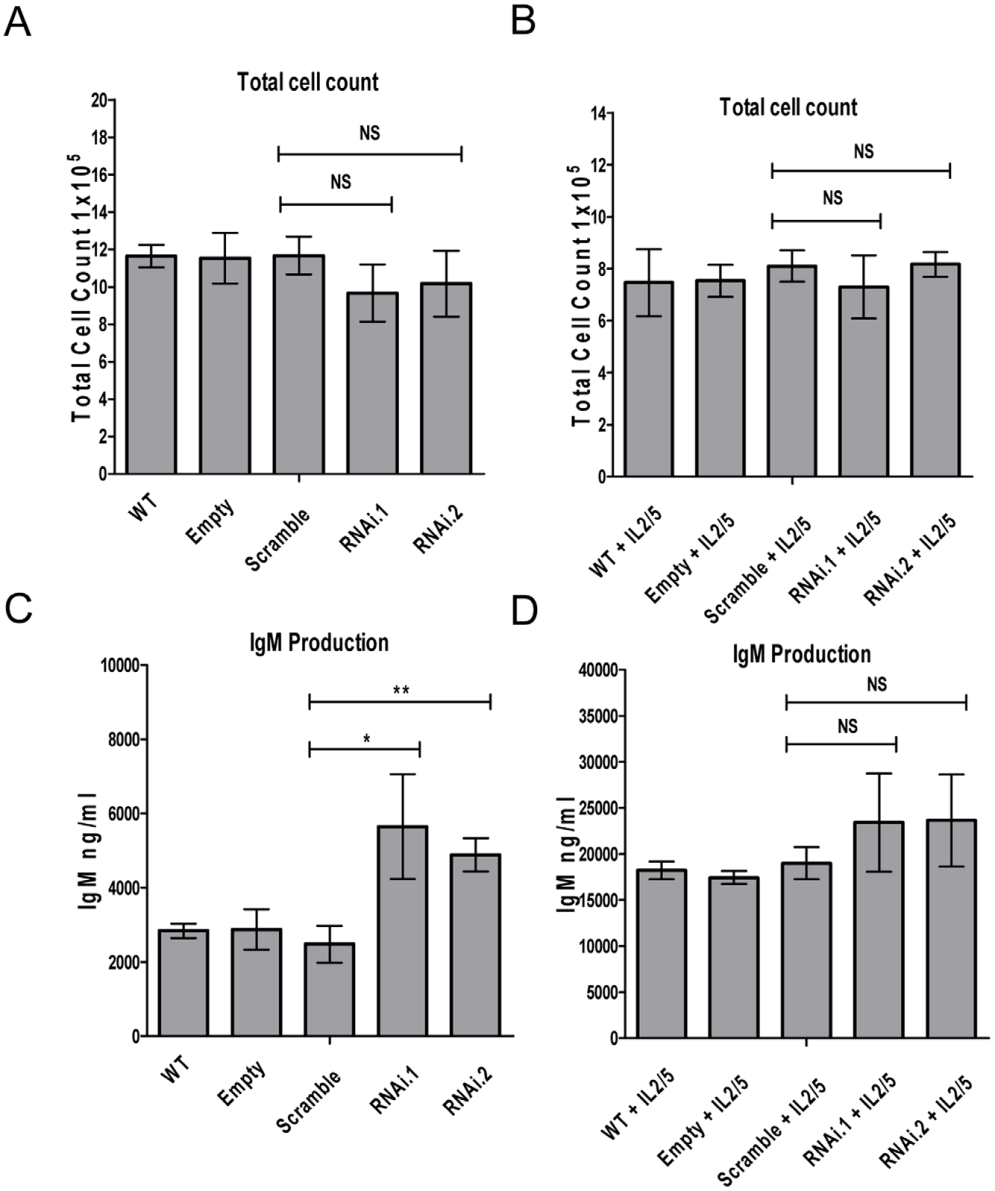
Lentiviral-mediated knockdown of ZFP36L1 expression in BCL1 cells promotes plasmacytoid differentiation by inducing IgM production. Cell numbers in the absence (A) and presence (B) of cytokines (IL-2 and IL-5) in pSicoR.zfp36l1.RNAi1 and pSicoR.zfp36l1.RNAi2 BCL1 cells compared to compared to wild-type, empty vector or pSicoR.scramble.RNAi infected cells. Cells numbers were counted in quadruplicate 4 days after seeding 2×10^5^/ml cells on day 0 in flasks in the absence or presence of cytokines (IL-2 20 ng/ml and IL-5 5 ng/ml). Mean ±SD (n = 3) are shown for three independent cell lines generated for each of the lentivirus infected cells. IgM secretion (ng/ml) measured by ELISA, 4 days after the start of the culture, in the absence (C) and presence (D) of cytokines (IL-2 20 ng/ml and IL-5 5 ng/ml) in pSicoR.zfp36l1.RNAi1 and pSicoR.zfp36l1.RNAi2 BCL1 cells compared to compared to wild-type, empty vector or pSicoR.scramble.RNAi infected cells. Mean ±SD (n = 3) are shown for three independent cell lines generated for each of the lentivirus infected cells. ** = p<0.01, * = p<0.05 as determined by t-test.

To further investigate the role of ZFP36L1 in proliferation and differentiation of BCL1 cells, lentivirus constructs expressing two different zfp36l1 shRNAs (pSicoR.zfp36l1.RNAi1 and pSicoR.zfp36l1.RNAi2) were designed to target and knock-down expression of ZFP36L1. As shown in Fig. 4, transduction with both pSicoR.zfp36l1.RNAi1 and pSicoR.zfp36l1.RNAi2 was effective in down-regulating endogenous ZFP36L1 in BCL1 cells at both protein and mRNA levels (Fig. 4A and B). pSicoR.zfp36l1.RNAi-transduced BCL1 cells proliferated less than wild-type, scramble or empty vector pSicoR virus control BCL1 cells in the absence of cytokines although this effect did not reach statistical significance (Fig. 5A). Similarly, no significant effect on cell proliferation was seen following cytokine induction in pSicoR.zfp36l1.RNAi-transduced cells (Fig. 5B). However, knock-down of ZFP36L1 induced a significant increase in IgM production in the absence of cytokines (Fig. 5C) and a more modest increase was seen in the presence of cytokines IL-2/5 which did not reach statistical significance (Fig. 5D). These observations indicate that loss of ZFP36L1 expression promotes plasmacytoid differentiation of BCL1 cells.

### Reconstruction of the Gene Regulatory Network of ZFP36L1 in B cells

**Figure 6:**
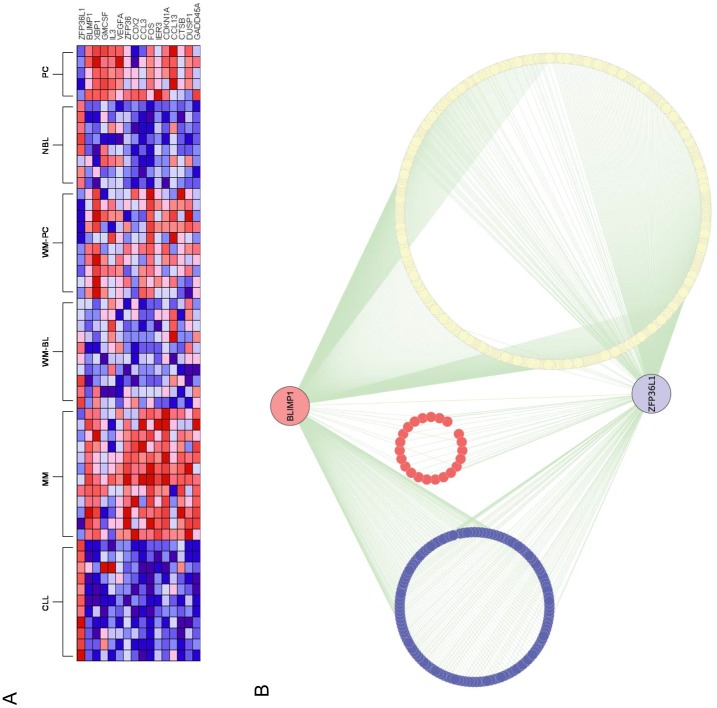
Microarray expression profile and ARACNe network of ZFP36L1. (A) Heat map expression profile of known ZFP36L1 target genes in terminal B cell differentiation. The profiles are compared with the reference genes, ZFP36L1, BLIMP1 and XBP1 (first three rows). GMCSF, VEGFA and IL-3 are validated ZFP36L1 targets; the remaining genes have been validated for ZFP36 [4]. Data was taken from GEO Accession number GSE 6691 [29]. Red indicates high and blue, low expression. Key: CLL: B chronic lymphocytic leukemia, MM: multiple myeloma, WM-BL: Waldenstrom’s macroglobulinemia B cells, WM-PB: Waldenstrom’s macroglobulinemia plasma cells, NBC: normal B cells, PC: normal plasma cells. (B) Graphical representation of the ARACNe network for BLIMP1 and ZFP36L1. Nodes representing the BLIMP1 and ZFP36L1 hubs are shown enlarged. Only first neighbours of these hubs are shown in the module. Nodes representing inferred BLIMP1 targets (significantly up-regulated in normal B cells) are blue; nodes representing inferred ZFP36L1 targets (significantly up-regulated in normal plasma cells) are red. Network graphics were generated as group attribute layout using Cytoscape version 2.6.0. [35].

To investigate the molecular mechanisms through which the functions of ZFP36L1 are integrated in the gene regulatory programme orchestrating B cell differentiation, we performed data-mining of a large microarray gene expression dataset [29] representing primary normal and malignant mature B and plasma cells. As shown in Fig. 6A, the expression profile of ZFP36L1 parallels that seen in the various B cell lines (Fig. 1) with high expression in cells of B cell phenotype and low expression in normal/malignant plasma cells. Significantly, the expression profile of ZFP36L1 was reciprocal to that of the B cell transcription factors, BLIMP1 and XBP1, that are known to function as key regulators of plasma cell differentiation [37], [38]. Several other genes whose mRNAs are well-characterised targets of ZFP36 family members were also expressed in a reciprocal pattern to that of ZFP36L1 (Fig. 6A) suggesting that they may also be targeted by ZFP36L1 in B cells, but not in plasma cells where ZFP36L1 is down-regulated.

Interestingly, we also observed that the expression pattern of the ZFP36L1 gene is reciprocal to that of ZFP36 (Fig. 6A) suggesting that the ZFP36 protein may target a distinct set of mRNAs that are down-regulated in plasma cells. To explore this, we examined the expression profile of a 393 gene signature representing mRNAs that have been identified as direct ZFP36 targets by immunoprecipitation and Affymetrix GeneChip analysis in dendritic cells [40]. Fig. S2 shows a heat map expression profile of these genes in the microarray dataset. At least in normal B lymphocytes and plasma cells, most of these genes are down-regulated in plasma cells relative to normal B cells (a reciprocal expression pattern to that of ZFP36– see Fig. 6). Indeed, genes that display significant down-regulation in normal plasma relative to normal B cells were significantly over-represented in the 393 gene signature (P = <0.0001 by Chi-square test). This was confirmed by Gene Set Enrichment Analysis (Fig. S3). It therefore seems highly likely that many of mRNAs encoded by these genes are targeted by ZFP36 (but not by ZFP36L1) in plasma cells.

To more objectively identify candidate mRNA targets for ZFP36L1 at a global level in mature B cells and to investigate how the functions of this protein are integrated in the plasmacytoid differentiation programme, the ARACNe algorithm (algorithm for the reconstruction of accurate gene regulatory cellular networks) [32], [33] was used to reconstruct a gene regulatory network using the microarray dataset used in Fig. 6A [29]. As shown in Fig. 6B, ZFP36L1 forms part of a module that is intimately associated with BLIMP1. In addition to being directly connected, the ZFP36L1 and BLIMP1 hubs share numerous first neighbours in the network (a spring-embedded graphic layout of the network is shown in Fig. S4). In order to reduce the incidence of ‘false-positives’ of inferred gene targets, we applied a stringent filter of significant up-regulation in normal B cells for BLIMP1 and of significant up-regulation in normal plasma cells for ZFP36L1. This approach was validated by determining whether the resulting list of 138 inferred BLIMP1 targets (Table S2) was significantly enriched for known (experimentally validated) targets for this transcription factor. The BLIMP1-inferred targets accounted for 16 out of a list of 64 genes (25%) that are negatively regulated by BLIMP1 in plasma cell differentiation in experimental studies [37], [41]. This figure increases to 25 genes (39%) when all inferred BLIMP1 targets are considered without applying the filter of significant up-regulation in normal B cells, but only at the expense of a lower enrichment score (data not shown). Overall, 11.6% of the 138 inferred BLIMP1 targets (Table S2) represent known targets for this transcription factor. Although relatively modest, this level of enrichment is highly statistically significant (P = <0.0001 by Chi-square test). It should also be born in mind that the full set of genes that is repressed by BLIMP1 in plasma cells is likely to far exceed the 64 genes that have been experimentally validated in earlier studies [37], [41].

When ZFP36L1-inferred targets were filtered for significant up-regulation in plasma cells, the resulting list comprised just 23 candidate target mRNAs (Table S3); the top-ranked scores for enrichment of Gene Ontology terms of this gene list are shown in Table S4. The proteins encoded by genes such as GAS6 (growth arrest) and GFI1 (transcriptional repression) –Table S4, would be expected to perform important roles in the plasma cell differentiation programme. Significantly, the list also included BLIMP1 itself which, together with CD38 and SEL1L encode mRNAs containing canonical 3′ ARE elements that are targeted by the ZFP36 protein family [4]. The absence of such regulatory elements in mRNAs of the remaining 20 genes is not unexpected; previous studies have shown that only a small minority of mRNAs that are functionally targeted by the related protein, ZFP36, contain AREs [4], [40]. Although none of the previously well-characterised ZFP36 family targets shown in Fig. 6A were represented in the ARACNe network shown in Fig. 6B, most of these genes were found to be directly connected to ZFP36L1 in ARACNe networks constructed using lower MI and higher DPI tolerance thresholds, but only by incurring an unacceptable false-positive rate (data not shown).

### Blimp1 mRNA Level is Regulated by ZFP36L1

**Figure 7:**
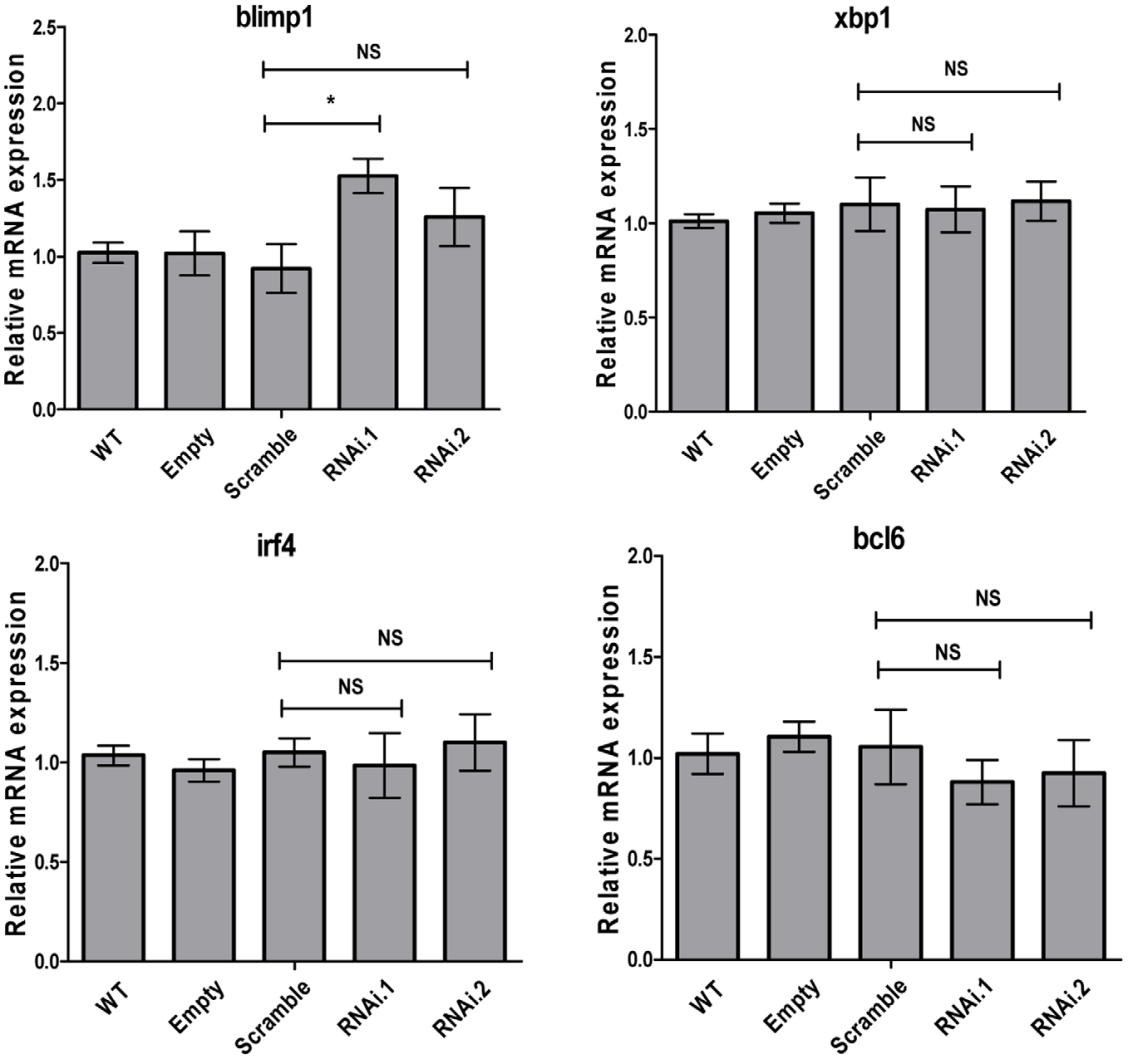
qRT-PCR analysis of plasmacytoid differentiation associated mRNAs in different BCL1 cell lines. qRT-PCR analysis of blimp1, xbp1, irf4 and bcl6 mRNA expression in pSicoR.zfp36l1.RNAi1 and pSicoR.zfp36l1.RNAi2 BCL1 cells compared to compared to wild-type, empty vector or pSicoR.scramble.RNAi infected cells. Cells were cultured in medium alone for 48h. Total RNA was extracted from 5×10^6^ cells, 1 µg RNA was reverse transcribed and the resulting cDNA was used as template for qRT-PCR assay with mouse gene specific primers for blimp1, xbp1, irf4 and bcl6. The 2^–ΔΔCT^ method of relative quantification was used to determine the fold change in mRNA expression compared to levels in wild-type cells. Mean ±SD (n = 3) are shown for three independent cell lines generated for each of the lentivirus infected cells. * = p<0.05 as determined by t-test.

**Figure 8:**
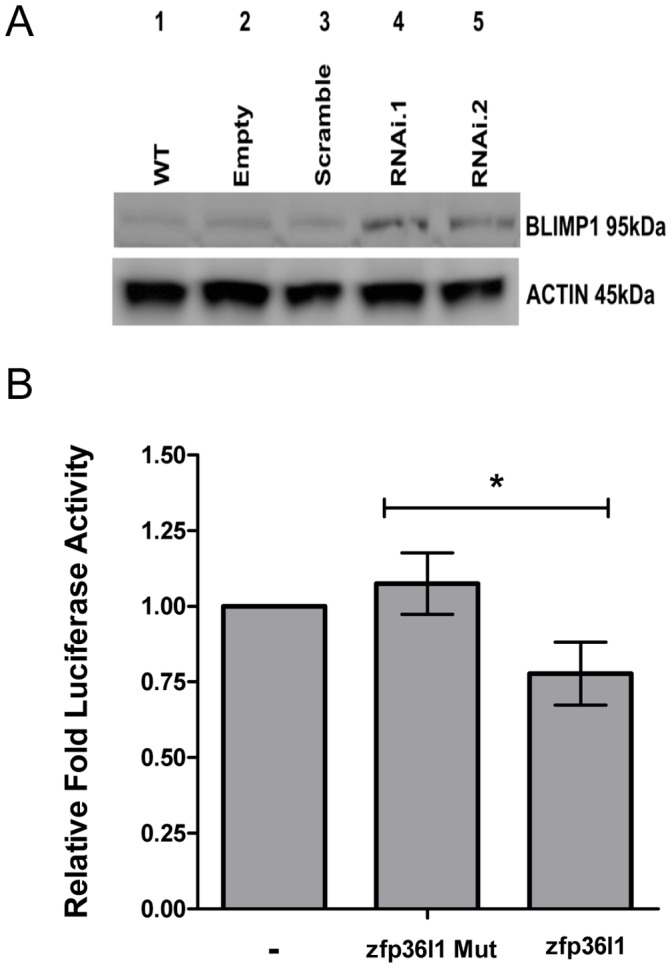
ZFP36L1 knockdown in BCL1 cells is associated with BLIMP1 upregulation and ZFP36L1 interacts with the BLIMP 3′UTR. (A) Western blot analysis of BLIMP1 levels in control and ZFP36L1 knockdown cells. BLIMP1 expression levels are upregulated in ZFP36L1 knockdown cells compared to controls. βACTIN levels are shown as a loading control. (B) ZFP36L1 mediates degradation of the BLIMP1 3′UTR. HEK 293 T cells were transfected with pMIRBLIMP1 3′UTR construct alone (control) or with either ZFP36L1 or a zinc finger domain mutant, ZFP36L1 Mut. Renilla luciferase was also included in all transfections as a normalization control. 24 hours later cell lysates were harvested and firefly and renilla luciferase levels measured using a Fluorstar Optima plate reader. Relative levels of luciferase activity were measured. Mean ±SD, are shown for four independent experiments (n = 4), * = p<0.05 as determined by t-test.

To experimentally validate mRNA targets for ZFP36L1 that are functionally important in regulating B cell differentiation, we focussed on BLIMP1, inferred from ARACNe analysis above, together with three other key transcriptional regulators of plasma cell differentiation, XBP1, IRF4 and BCL6 [37]–[39]. As shown in Fig. 7, the expression of xbp1, irf4 and bcl6 mRNAs were not significantly affected in pSicoR.zfp36l1.RNAi transduced BCL1 cells compared to control cells in the absence of cytokine induction. By contrast, there was a significant increase in levels of blimp1 mRNA seen before cytokine treatment in pSicoR.zfp36l1.RNAi1-transduced BCL1 cells compared to controls, with a smaller increase in pSicoR.zfp36l1.RNAi2-transduced BCL1 cells (Fig. 7). There was also a trend towards higher blimp1 mRNA levels that did not reach statistical significance in the presence of cytokines (data not shown). Importantly, pSicoR.zfp36l1.RNAi-transduced BCL1 cells expressed higher levels of BLIMP1 protein compared to control cells (Fig. 8A). Finally, a functional effect of ZFP36L1 on the BLIMP1 3′UTR sequence was demonstrable by luciferase reporter assay in which wild-type ZFP36L1 significantly reduced BLIMP1 3′UTR-driven luciferase levels compared to a zinc finger mutant version of ZFP36L1 (Fig. 8B).

## Discussion

Accumulating evidence implicates the mRNA destabilisation protein, ZFP36L1, in the regulation of self-renewal and differentiation in cells of diverse lineages including haematopoietic cells [5]. In mouse embryonic stem (ES) cells, high levels of ZFP36L1 reportedly maintain an undifferentiated state with down-regulation inducing differentiation to cardiomyocytes [20]. Similarly, in CD34+ hematopoietic stem cells, ZFP36L1 negatively regulates erythroid differentiation [42]. ZFP36L1 has also been found to be overexpressed in cell lines and primary cells expressing the AML1-ETO fusion protein that is found in 40% of Acute Myeloid Leukaemia of M2 sub-type. Enforced over-expression of ZFP36L1 promotes proliferation and inhibits differentiation of these cells [19]. Finally, in mouse thymocytes, targeted deletion of the *ZFP36L1* and *ZFP36L2* genes leads to a perturbation of NOTCH-1-regulated thymic development *in vivo* with accumulation of T cell antigen receptor β-chain-negative cells culminating in T lymphoblastic leukaemia [43].

The results from our study implicate ZFP36L1 in playing a key role in B lymphopoiesis by regulating the transition from mature B cell to plasma cell. In cell line models and murine primary B cells, ZFP36L1 expression was high in B lymphocytes, but largely extinguished in plasma cells. Enforced expression of ZFP36L1 in the mouse BCL1 cell line blocked cytokine-induced differentiation while shRNA-mediated knock-down of this gene enhanced cytokine-induced differentiation. These observations are consistent with ZFP36L1 being required to negatively regulate terminal plasmacytoid differentiation.

By using an information-theoretic method for reconstruction of gene regulatory networks (ARACNe) [32], [33], we inferred a direct regulatory interaction between ZFP36L1 and the plasma cell transcription factor, BLIMP1. shRNA-mediated knock-down of ZFP36L1 in BCL1 cells led to a modest, but significant increase in blimp1 mRNA and protein levels. The mRNA expression levels of other key transcriptional regulators of plasmacytoid differentiation (xbp1, irf4, bcl6) [38], [39] was unaffected by ZFP36L1 knock-down. In further experiments, we have similarly observed no effect of ZFP36L1 knock-down on mRNA levels of pax5 (data not shown). Since BLIMP1 mRNA harbours a canonical AU-rich recognition motif in its 3′UTR, we hypothesised that it was highly likely that ZFP36L1 directly regulates BLIMP1 expression by targeting its mRNA and we found evidence for this in BLIMP 3′ UTR luciferase reporter assays. The elevated levels of blimp1 mRNA and protein seen in ZFP36L1-knock-down BCL1 cells could at least in part explain the enhanced differentiation seen in these cells as evidenced by increased IgM secretion. Interestingly, ZFP36L1 has previously been identified as part of a signature of genes that is repressed by BLIMP1 in B cells during the transition of mature B cells to plasma cells [37], [41]. More recently, ZFP36L1 has been identified as a direct BLIMP1 target gene in B-lineage cells by chromatin immunoprecipitation analysis [44]. Thus, ZFP36L1 and BLIMP1 may exist in a regulatory feedback loop where ZFP36L1 suppresses premature/leaky BLIMP1 expression in B cells; in response to triggers of differentiation, accumulating levels of BLIMP1 would then transcriptionally silence ZFP36L1 expression, allowing plasma cell differentiation to proceed.

ARACNe analysis also identified a number of other candidate mRNA targets of ZFP36L1. Whether these are targeted via their 3′ UTRs and their roles in regulating plasmacytoid differentiation remains to be determined. However, several well-characterised/validated targets of ZFP36 family members were identified that displayed a reciprocal pattern of expression to ZFP36L1. Most notable amongst these were four genes for which there is direct experimental evidence for their roles in regulating B cell differentiation, Fos [45], GMCSF, IL3 [46] and COX2 [47]. In further experiments, we observed no effect of anti-GMCSF antibody on IL-2/IL-5-induced differentiation of BCL1 cells (J Murphy, unpublished observations). However, given that ZFP36L1 likely targets mRNAs of multiple genes that act cooperatively to orchestrate the transition from B-cells to plasma cells, the value of such ‘single candidate gene’ experiments is questionable. Finally, we noted a reciprocal pattern of expression between ZFP36L1 and ZFP36 in cells of B lymphocyte and plasma cell phenotype. Moreover, genes that displayed significant down-regulation in normal plasma cells relative to normal B cells were significantly over-represented in ZFP36 targets that have previously been validated in dendritic cells [40]. Given the mutually exclusive expression patterns of their genes, it follows that the ZFP36L1 and ZFP36 proteins would target distinct sets of mRNAs during the transition of B-cell to plasma cells.

## Supporting Information

Figure S1 ZFP36L1 and ZFP36 expression in B cells. Immunohistological staining of tonsil germinal centres with (A) anti-ZFP36L1 antibody, (B) with control rabbit serum. Sections are shown (40X magnification) after counterstaining with haemotoxylin. Arrows depict the mantle zone region surrounding a germinal centre. (C) Western blot showing ZFP36 expression in IL-2/5 treated murine leukemic BCL1 cells. Protein lysates were made from cells 96 h after stimulation with cytokines (20 ng/ml IL-2 and 5 ng/ml IL-5) (lane 1) or from unstimulated cells (lane 2). ZFP36 and HSP90 proteins were detected by anti-ZFP36 and anti-HSP90 antibodies respectively.
(TIF)

Figure S2 Heat map expression profile of ZFP36 dendritic cell target genes in terminal B cell differentiation. The profiles of 393 mRNAs that have been identified as ZFP36 targets by immunoprecipitation and Affymetrix GeneChip analysis in dendritic cells [40] is shown. The majority of these mRNAs that are expressed in normal B lymphocytes are down-regulated in normal plasma cells, consistent with them being targets for ZFP36. Data was taken from GEO Accession number GSE 6691 [29]. Red indicates high and blue, low expression. Key: CLL: B chronic lymphocytic leukemia, MM: multiple myeloma, WM-BL: Waldenstrom’s macroglobulinemia B cells, WM-PB: Waldenstrom’s macroglobulinemia plasma cells, NBC: normal B cells, PC: normal plasma cells.
(TIF)

Figure S3 Genes that are down-regulated in normal plasma cells *vs* B cells are enriched in a set of validated ZFP36 target genes. Gene set enrichment analysis (GSEA) [48] according to up-regulation (signal-to-noise test statistic) in normal B cells relative to plasma cells is shown as a profile of running Kolmogorov-Smirnov ES score and positions of ZFP36 marker genes (black bars) in the rank ordered list of differentially expressed genes. The normalised enrichment score was 1.72 (P = <0.00001) corrected for 1000 gene permutation tests. Of the 371 unique ZFP36 target genes [40], annotated in GSEA, 200 are represented in the leading edge.
(TIF)

Figure S4 Graphical representation of the ARACNe network for BLIMP1 and ZFP36L1. Nodes representing the BLIMP1 and ZFP36L1 hubs are shown enlarged. Only first neighbours of these hubs are shown in the module. Nodes representing inferred BLIMP1 targets (significantly up-regulated in normal B cells) are blue; nodes representing inferred ZFP36L1 targets (significantly up-regulated in normal plasma cells) are red. Network graphics (spring-embedded layout) were generated using Cytoscape version 2.6.0.
(TIF)

Table S1 Oligonucleotide primers designed for construction of zfp36l1 shRNA and scramble sequences.
(DOC)

Table S2 BLIMP1 target genes inferred from ARACNe analysis. The Bonferroni-corrected P values (P = ≤0.05) for the significance of up-regulation in normal B cells are shown. Known BLIMP targets were taken from the SignatureDB [37], [41].
(DOC)

Table S3 ZFP36L1 targets inferred from ARACNe analysis. Values of the false discovery rate (FDR = ≤0.01) for enrichment in normal plasma cells are shown. Data on the presence and type of ARE element were taken from the ARED database [49].
(DOC)

Table S4 Enrichment of Gene Ontology terms for ARACNe-inferred ZFP36L1 targets.
(DOC)
