# Supplementary figures and images for: ZFP36L1 Negatively Regulates Plasmacytoid Differentiation of BCL1 Cells by Targeting BLIMP1 mRNA
Source: PLoS One. 2012 Dec 20;7(12):e52187. doi: 10.1371/journal.pone.0052187 (PMC3527407; doi:10.1371/journal.pone.0052187)

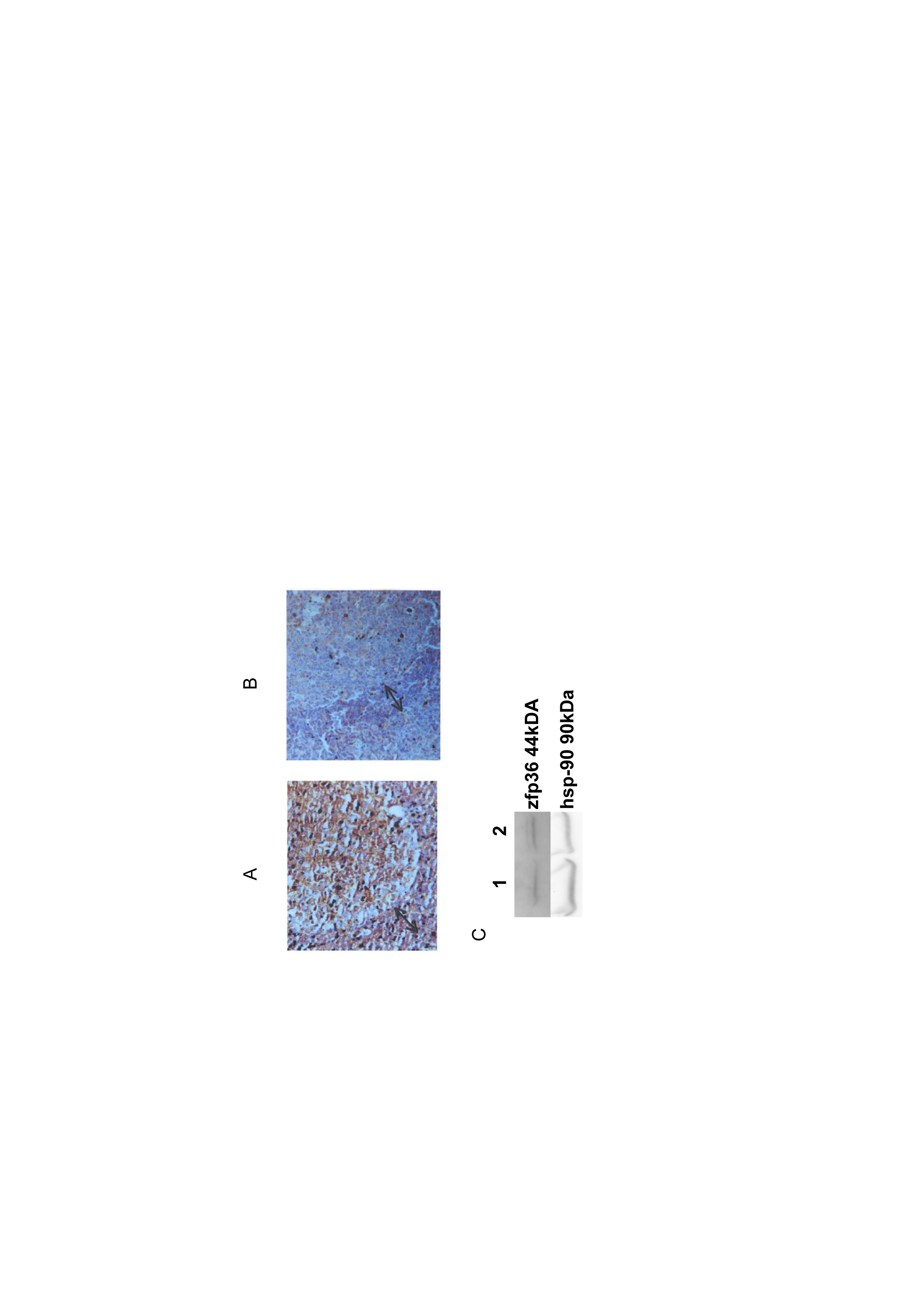

Supplement: Figure S1 — ZFP36L1 and ZFP36 expression in B cells. Immunohistological staining of tonsil germinal centres with (A) anti-ZFP36L1 antibody, (B) with control rabbit serum. Sections are shown (40X magnification) after counterstaining with haemotoxylin. Arrows depict the mantle zone region surrounding a germinal centre. (C) Western blot showing ZFP36 expression in IL-2/5 treated murine leukemic BCL1 cells. Protein lysates were made from cells 96 h after stimulation with cytokines (20 ng/ml IL-2 and 5 ng/ml IL-5) (lane 1) or from unstimulated cells (lane 2). ZFP36 and HSP90 proteins were detected by anti-ZFP36 and anti-HSP90 antibodies respectively. (TIF) [file pone.0052187.s001.tif]

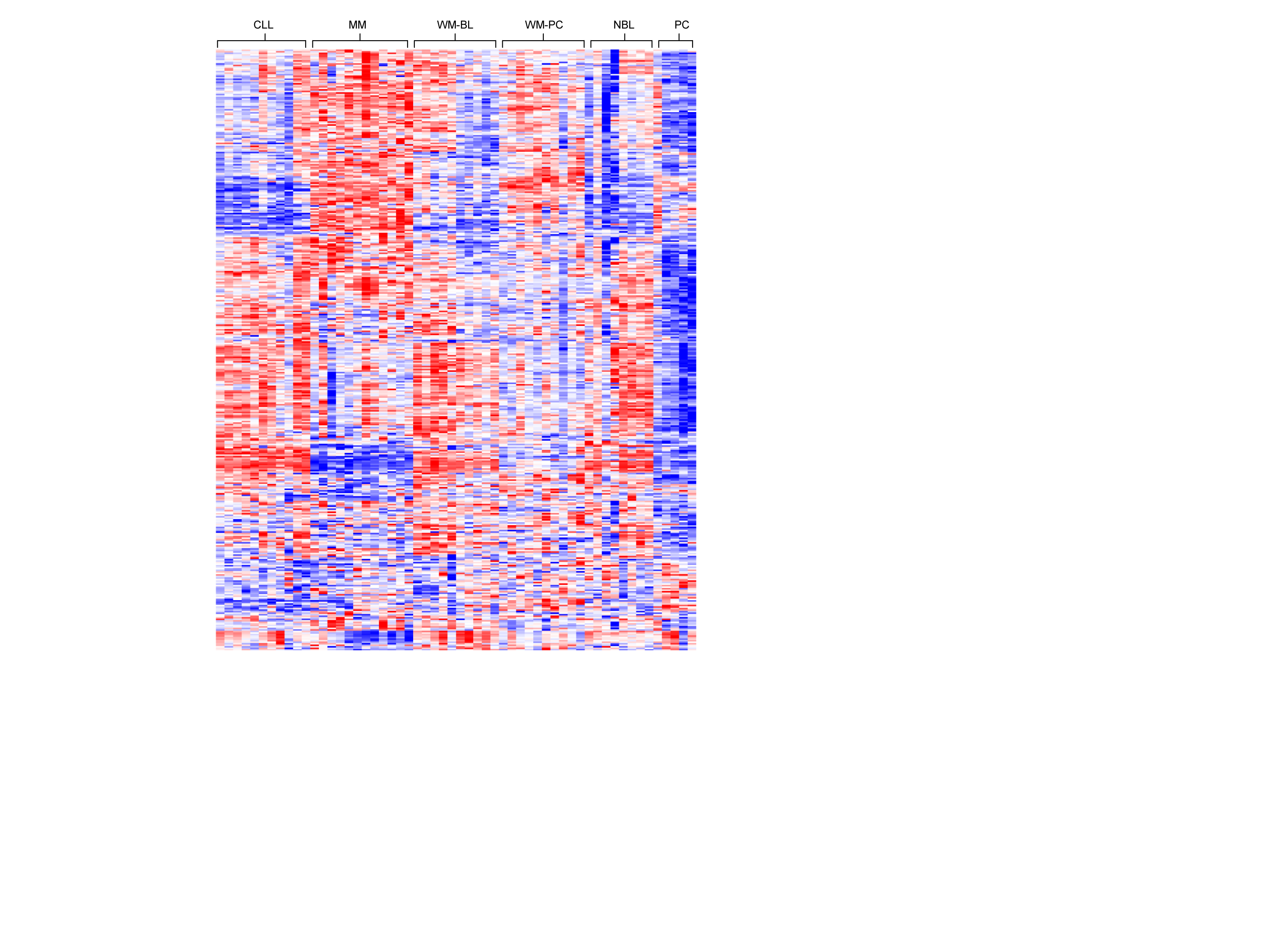

Supplement: Figure S2 — Heat map expression profile of ZFP36 dendritic cell target genes in terminal B cell differentiation. The profiles of 393 mRNAs that have been identified as ZFP36 targets by immunoprecipitation and Affymetrix GeneChip analysis in dendritic cells [40] is shown. The majority of these mRNAs that are expressed in normal B lymphocytes are down-regulated in normal plasma cells, consistent with them being targets for ZFP36. Data was taken from GEO Accession number GSE 6691 [29]. Red indicates high and blue, low expression. Key: CLL: B chronic lymphocytic leukemia, MM: multiple myeloma, WM-BL: Waldenstrom’s macroglobulinemia B cells, WM-PB: Waldenstrom’s macroglobulinemia plasma cells, NBC: normal B cells, PC: normal plasma cells. (TIF) [file pone.0052187.s002.tif]

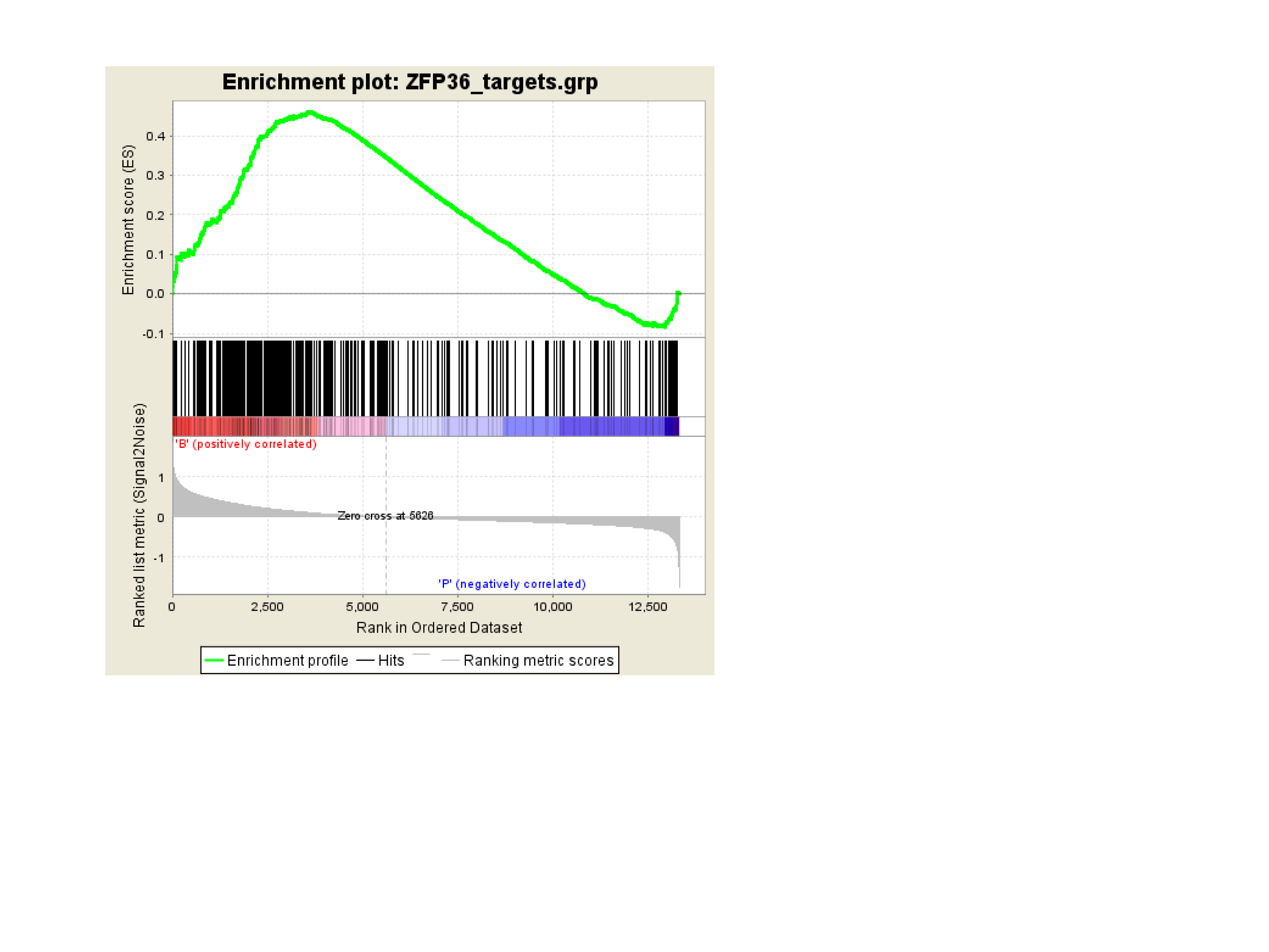

Supplement: Figure S3 — Genes that are down-regulated in normal plasma cells vs B cells are enriched in a set of validated ZFP36 target genes. Gene set enrichment analysis (GSEA) [48] according to up-regulation (signal-to-noise test statistic) in normal B cells relative to plasma cells is shown as a profile of running Kolmogorov-Smirnov ES score and positions of ZFP36 marker genes (black bars) in the rank ordered list of differentially expressed genes. The normalised enrichment score was 1.72 (P = <0.00001) corrected for 1000 gene permutation tests. Of the 371 unique ZFP36 target genes [40], annotated in GSEA, 200 are represented in the leading edge. (TIF) [file pone.0052187.s003.tif]

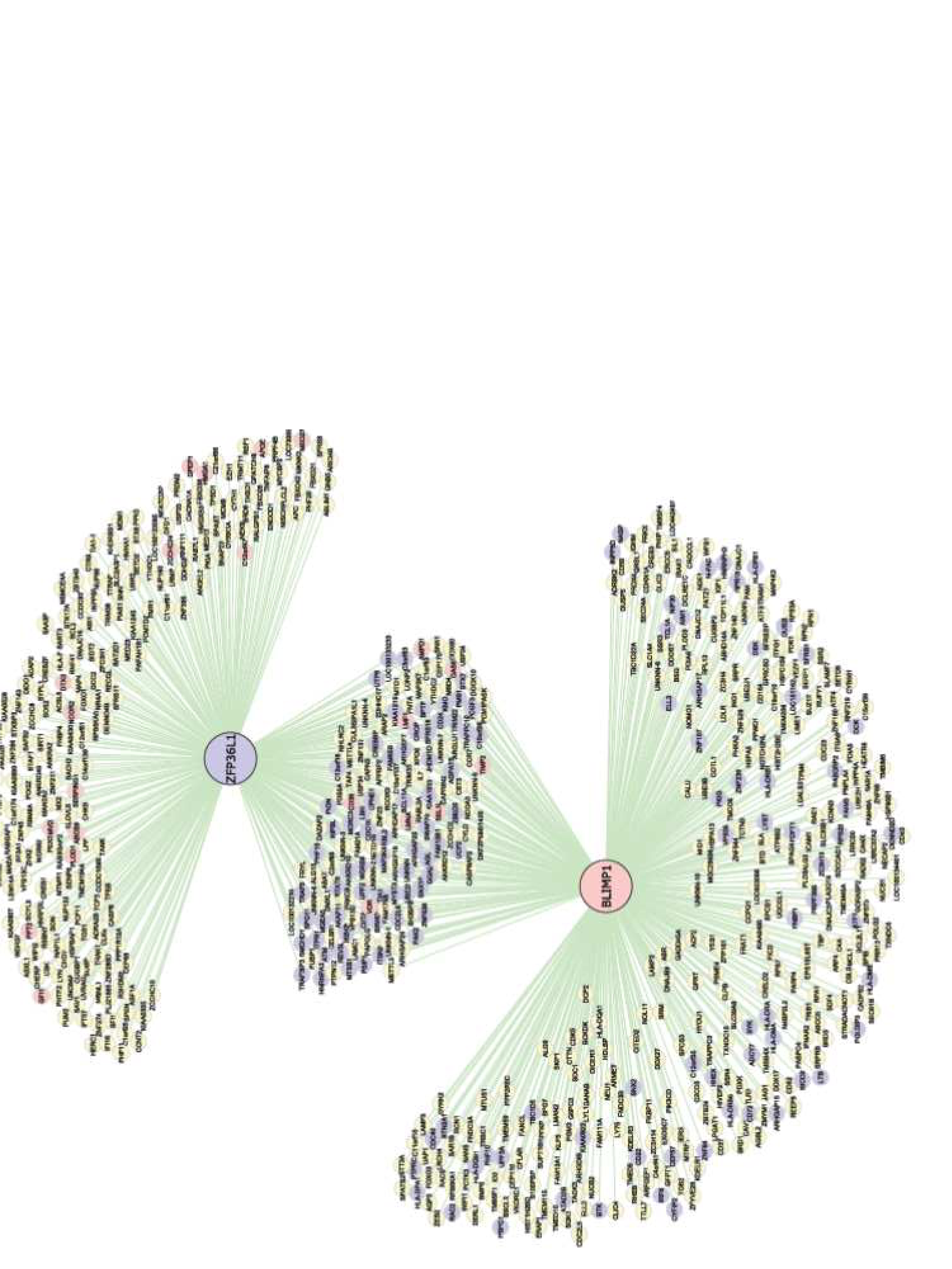

Supplement: Figure S4 — Graphical representation of the ARACNe network for BLIMP1 and ZFP36L1. Nodes representing the BLIMP1 and ZFP36L1 hubs are shown enlarged. Only first neighbours of these hubs are shown in the module. Nodes representing inferred BLIMP1 targets (significantly up-regulated in normal B cells) are blue; nodes representing inferred ZFP36L1 targets (significantly up-regulated in normal plasma cells) are red. Network graphics (spring-embedded layout) were generated using Cytoscape version 2.6.0. (TIF) [file pone.0052187.s004.tif]
