# Supplementary material for: ZFP36L1 Negatively Regulates Plasmacytoid Differentiation of BCL1 Cells by Targeting BLIMP1 mRNA
Source: PLoS One. 2012 Dec 20;7(12):e52187. doi: 10.1371/journal.pone.0052187 (PMC3527407; doi:10.1371/journal.pone.0052187)
Supplement: Table S1 — Oligonucleotide primers designed for construction of zfp36l1 shRNA and scramble sequences. (DOC) [file pone.0052187.s005.doc]

**Table S1.** Oligonucleotide primers designed for construction of zfp36l1 shRNA and scramble sequences. Note zfp36l1 shRNA sequences shown target both human and mouse zfp36l1 mRNAs.

| Primer Name | Primer Sequence  5’-3’ | Length  bp | Modification |
| --- | --- | --- | --- |
| zfp36l1.RNAi.1.F | 5’TGTAACAAGATGCTCAACT ATTCAAGAGATAGTTGAGC ATCTTGTTACTTTTTTC 3’ | 55 | 5’ Phosphate |
| zfp36l1.RNAi.1.R | 5’TCGAGAAAAAAGTAACAAG ATGCTCAACTATCTCTTGAA TAGTTGAGCATCTTGTTACA3’ | 59 | 5’ Phosphate |
| zfp36l1.RNAi.2.F | 5’TGCAACTTAGTGCCTTGTAA TTCAAGAGATTACAAGGCA CTAAGTTGCTTTTTTC3’ | 55 | 5’ Phosphate |
| zfp36l1.RNAi.2.R | 5’TCGAGAAAAAAGCAACTTA GTGCCTTGTAATCTCTT GAA TTACAAGGCACTAAGTTGCA3’ | 59 | 5’ Phosphate |
| scramble.RNAi.1.F | 5’TGAACTCAAGACCGATATTA TTCAAGAGATAATATCGG TC TTGAGTTCTTTTTTC3’ | 55 | 5’ Phosphate |
| scramble.RNAi.1.R | 5’TCGAGAAAAAAGAACTCAA GACCGATATTATCTCTTGAA TAATATCGGTCTTGAGTTCA3’ | 59 | 5’ Phosphate |
