# Supplementary material for: ZFP36L1 Negatively Regulates Plasmacytoid Differentiation of BCL1 Cells by Targeting BLIMP1 mRNA
Source: PLoS One. 2012 Dec 20;7(12):e52187. doi: 10.1371/journal.pone.0052187 (PMC3527407; doi:10.1371/journal.pone.0052187)
Supplement: Table S2 — BLIMP1 target genes inferred from ARACNe analysis. The Bonferroni-corrected P values (P = ≤0.05) for the significance of up-regulation in normal B cells are shown. Known BLIMP targets were taken from the SignatureDB [37], [41]. (DOC) [file pone.0052187.s006.doc]

**Table S2.** **BLIMP1 target genes inferred from ARACNe analysis.** The Bonferroni-corrected P values (P = ≤ 0.05) for the significance of up-regulation in normal B cells are shown. Known BLIMP targets were taken from the SignatureDB

| AffyID | Entrez | Gene Symbol | P value | Known target |
| --- | --- | --- | --- | --- |
| 213293_s_at | 10346 | TRIM22 | 1.42E-05 | no |
| 209306_s_at | 23075 | SWAP70 | 6.21E-05 | no |
| 44790_s_at | 80183 | C13orf18 | 4.78E-06 | no |
| 213142_x_at | 54103 | PION | 8.83E-06 | no |
| 218715_at | 55813 | UTP6 | 9.30E-06 | no |
| 221969_at | UNKNN | UNKNN | 1.30E-05 | no |
| 212098_at | 151162 | LOC151162 | 1.79E-05 | no |
| 221601_s_at | 9214 | FAIM3 | 2.47E-05 | no |
| 212632_at | 8417 | STX7 | 3.02E-05 | no |
| 212288_at | 23048 | FNBP1 | 4.27E-05 | no |
| 217896_s_at | 80011 | NIP30 | 4.88E-05 | no |
| 203037_s_at | 9788 | MTSS1 | 5.13E-05 | no |
| 221011_s_at | 81606 | LBH | 5.26E-05 | no |
| 209341_s_at | 3551 | IKBKB | 5.65E-05 | no |
| 202160_at | 1387 | CREBBP | 6.73E-05 | no |
| 208894_at | 3122 | HLA-DRA | 7.03E-05 | no |
| 209829_at | 9750 | FAM65B | 7.94E-05 | no |
| 204057_at | 3394 | IRF8 | 9.95E-05 | yes |
| 209659_s_at | 8881 | CDC16 | 1.11E-04 | no |
| 200934_at | 7913 | DEK | 1.59E-04 | yes |
| 205307_s_at | 8564 | KMO | 1.86E-04 | no |
| 205804_s_at | 80342 | TRAF3IP3 | 2.50E-04 | no |
| 200899_s_at | 10724 | MGEA5 | 2.72E-04 | no |
| 214369_s_at | 10235 | RASGRP2 | 2.80E-04 | no |
| 210347_s_at | 53335 | BCL11A | 2.94E-04 | no |
| 208760_at | 7329 | UBE2I | 3.59E-04 | no |
| 218870_at | 55843 | ARHGAP15 | 4.53E-04 | no |
| 203513_at | 80208 | SPG11 | 4.54E-04 | no |
| 203932_at | 3109 | HLA-DMB | 4.61E-04 | yes |
| 212543_at | 202 | AIM1 | 4.92E-04 | no |
| 212176_at | 25957 | SFRS18 | 5.00E-04 | no |
| 213666_at | 23157 | UNKNN | 5.75E-04 | no |
| 211991_s_at | 3113 | HLA-DPA1 | 6.39E-04 | yes |
| 39318_at | 8115 | TCL1A | 6.49E-04 | no |
| 204352_at | 7188 | TRAF5 | 6.57E-04 | yes |
| 218090_s_at | 55717 | BRWD2 | 6.64E-04 | no |
| 208914_at | 23062 | GGA2 | 6.77E-04 | no |
| 204689_at | 3087 | HHEX | 7.08E-04 | no |
| 207339_s_at | 4050 | LTB | 7.12E-04 | no |
| 207238_s_at | 5788 | PTPRC | 7.16E-04 | no |
| 208990_s_at | 3189 | HNRNPH3 | 8.55E-04 | no |
| 209337_at | 11168 | PSIP1 | 8.58E-04 | no |
| 209285_s_at | 23272 | C3orf63 | 9.15E-04 | no |
| 208998_at | 7351 | UCP2 | 0.001118 | no |
| 213387_at | 54454 | ATAD2B | 0.001276 | no |
| 215193_x_at | 3123 | HLA-DRB1 | 0.001375 | yes |
| 212774_at | 10472 | ZNF238 | 0.001408 | no |
| 202732_at | 11142 | PKIG | 0.001488 | no |
| 203332_s_at | 3635 | INPP5D | 0.001491 | yes |
| 204882_at | 9938 | ARHGAP25 | 0.001989 | no |
| 221081_s_at | 79961 | DENND2D | 0.002 | no |
| 213888_s_at | 80342 | LOC100133233 | 0.002202 | no |
| 205739_x_at | 51427 | ZNF107 | 0.002315 | no |
| 212414_s_at | 23157 | N-PAC | 0.00248 | no |
| 212402_at | 23091 | ZC3H13 | 0.002547 | no |
| 202548_s_at | 8874 | ARHGEF7 | 0.003008 | no |
| 207540_s_at | 6850 | SYK | 0.0031 | yes |
| 218505_at | 79726 | WDR59 | 0.003135 | no |
| 215785_s_at | 26999 | CYFIP2 | 0.003228 | no |
| 212331_at | 5934 | RBL2 | 0.003854 | no |
| 213154_s_at | 23299 | BICD2 | 0.003891 | no |
| 215933_s_at | 3087 | HHEX | 0.004248 | no |
| 221736_at | 57148 | KIAA1219 | 0.005154 | no |
| 209995_s_at | 8115 | TCL1A | 0.005192 | no |
| 203376_at | 51362 | CDC40 | 0.005897 | no |
| 208070_s_at | 5980 | REV3L | 0.005976 | no |
| 217362_x_at | 3128 | HLA-DRB6 | 0.006134 | no |
| 201137_s_at | 3115 | HLA-DPB1 | 0.00616 | yes |
| 203518_at | 1130 | LYST | 0.006732 | no |
| 209760_at | 23240 | KIAA0922 | 0.006776 | no |
| 204453_at | 7637 | ZNF84 | 0.007002 | no |
| 203302_at | 1633 | DCK | 0.007069 | no |
| 212660_at | 23338 | PHF15 | 0.007496 | no |
| 201814_at | 9779 | TBC1D5 | 0.007579 | no |
| 212100_s_at | 84271 | POLDIP3 | 0.008185 | no |
| 218263_s_at | 58486 | ZBED5 | 0.008625 | no |
| 203519_s_at | 26019 | UPF2 | 0.008626 | no |
| 212998_x_at | 3119 | HLA-DQB1 | 0.00867 | no |
| 200949_x_at | 6224 | RPS20 | 0.008809 | no |
| 218404_at | 29887 | SNX10 | 0.009403 | no |
| 217478_s_at | 3108 | HLA-DMA | 0.009452 | no |
| 203494_s_at | 9702 | CEP57 | 0.009823 | no |
| 212672_at | 472 | ATM | 0.010871 | no |
| 212469_at | 25836 | NIPBL | 0.010898 | no |
| 216379_x_at | 1.00E+08 | CD24 | 0.010921 | yes |
| 209412_at | 7109 | TRAPPC10 | 0.011569 | no |
| 202114_at | 6643 | SNX2 | 0.012002 | no |
| 211784_s_at | 6426 | SFRS1 | 0.012307 | no |
| 219126_at | 55274 | PHF10 | 0.01231 | no |
| 218518_at | 51306 | FAM13B1 | 0.013395 | no |
| 202423_at | 7994 | MYST3 | 0.013543 | no |
| 212795_at | 23325 | KIAA1033 | 0.013746 | no |
| 210279_at | 2841 | GPR18 | 0.013748 | yes |
| 218093_s_at | 55608 | ANKRD10 | 0.013748 | no |
| 211962_s_at | 677 | ZFP36L1 | 0.01426 | yes |
| 201448_at | 7072 | TIA1 | 0.014301 | no |
| 213256_at | 115123 | Mar-03 | 0.01497 | no |
| 203944_x_at | 11120 | BTN2A1 | 0.015085 | no |
| 201969_at | 4678 | NASP | 0.015771 | no |
| 203566_s_at | 178 | AGL | 0.01735 | no |
| 205101_at | 4261 | CIITA | 0.017436 | yes |
| 221778_at | 80853 | JHDM1D | 0.017516 | no |
| 213775_x_at | 27332 | ZNF638 | 0.019024 | no |
| 219518_s_at | 80237 | ELL3 | 0.019107 | no |
| 218076_s_at | 55114 | ARHGAP17 | 0.019461 | no |
| 212899_at | 23097 | CDC2L6 | 0.021724 | no |
| 214323_s_at | 65110 | UPF3A | 0.023027 | no |
| 218371_s_at | 55269 | PSPC1 | 0.024159 | no |
| 221937_at | UNKNN | UNKNN | 0.025432 | no |
| 203156_at | 11215 | AKAP11 | 0.025554 | no |
| 213603_s_at | 5880 | RAC2 | 0.026945 | no |
| 212577_at | 23347 | SMCHD1 | 0.027095 | no |
| 204581_at | 933 | CD22 | 0.027196 | yes |
| 211929_at | 220988 | HNRNPA3 | 0.027473 | no |
| 205504_at | 695 | BTK | 0.027495 | yes |
| 217727_x_at | 55737 | VPS35 | 0.028936 | no |
| 201331_s_at | 6778 | STAT6 | 0.030417 | yes |
| 212170_at | 8904 | CPNE1 | 0.030742 | no |
| 208877_at | 5062 | PAK2 | 0.030847 | no |
| 213000_at | 23515 | MORC3 | 0.031925 | no |
| 208647_at | 2222 | FDFT1 | 0.033083 | no |
| 218040_at | 55119 | PRPF38B | 0.034417 | no |
| 222103_at | 466 | ATF1 | 0.034456 | no |
| 209898_x_at | 50618 | ITSN2 | 0.034464 | no |
| 202808_at | 54838 | C10orf26 | 0.035681 | no |
| 215925_s_at | 971 | CD72 | 0.037321 | no |
| 203741_s_at | 113 | ADCY7 | 0.037856 | no |
| 203710_at | 3708 | ITPR1 | 0.040004 | no |
| 220044_x_at | 51747 | CROP | 0.041649 | no |
| 209271_at | 2186 | BPTF | 0.042641 | no |
| 47105_at | 54920 | DUS2L | 0.043116 | no |
| 204089_x_at | 4216 | MAP3K4 | 0.04338 | no |
| 212753_at | 10336 | PCGF3 | 0.043748 | no |
| 218614_at | 55196 | C12orf35 | 0.043797 | no |
| 218096_at | 55326 | AGPAT5 | 0.045368 | no |
| 213677_s_at | 5378 | PMS1 | 0.049319 | no |
