# Supplementary material for: ZFP36L1 Negatively Regulates Plasmacytoid Differentiation of BCL1 Cells by Targeting BLIMP1 mRNA
Source: PLoS One. 2012 Dec 20;7(12):e52187. doi: 10.1371/journal.pone.0052187 (PMC3527407; doi:10.1371/journal.pone.0052187)
Supplement: Table S3 — ZFP36L1 targets inferred from ARACNe analysis. Values of the false discovery rate (FDR = ≤0.01) for enrichment in normal plasma cells are shown. Data on the presence and type of ARE element were taken from the ARED database [49]. (DOC) [file pone.0052187.s007.doc]

**Table S3. ZFP36L1 targets inferred from ARACNe analysis**

Values of the false discovery rate (FDR = ≤ 0.01) for enrichment in normal plasma cells are shown. Data on the presence and type of ARE element were taken from the ARED database

| AffyID | Entrez | Gene symbol | Gene description | FDR | ARE |  |
| --- | --- | --- | --- | --- | --- | --- |
| 202064_s_at | 6400 | SEL1L | sel-1 suppressor of lin-12-like (C. elegans) | 2.85E-05 | class 2, cluster 4 | |
| 200827_at | 5351 | PLOD1 | procollagen-lysine 1, 2-oxoglutarate 5-dioxygenase 1 | 9.15E-05 | no |  |
| 205692_s_at | 952 | CD38 | CD38 molecule | 1.14E-04 | class 1, cluster 5 | |
| 214209_s_at | 23457 | ABCB9 | ATP-binding cassette, sub-family B (MDR/TAP), member 9 | 1.58E-04 | no |  |
| 219135_s_at | 64788 | LMF1 | lipase maturation factor 1 | 1.75E-04 | no |  |
| 1598_g_at | 100133684 /// 2621 | GAS6 | similar to growth arrest-specific 6; growth arrest-specific 6 | 2.56E-04 | no |  |
| 203167_at | 7077 | TIMP2 | TIMP metallopeptidase inhibitor 2 | 4.50E-04 | no |  |
| 212884_x_at | 348 | APOE | hypothetical LOC100129500; apolipoprotein E | 8.67E-04 | no |  |
| 206589_at | 2672 | GFI1 | growth factor independent 1 transcription repressor | 0.001037 | no |  |
| 204255_s_at | 7421 | VDR | vitamin D (1,25- dihydroxyvitamin D3) receptor | 0.001374 | no |  |
| 209826_at | 9374 | PPT2 | palmitoyl-protein thioesterase 2 | 0.001393 | no |  |
| 210457_x_at | 3159 | HMGA1 | hypothetical LOC100130009; high mobility group AT-hook 1 | 0.00176 | no |  |
| 221835_at | 196403 | DTX3 | deltex homolog 3 (Drosophila) | 0.001927 | no |  |
| 217192_s_at | 639 | BLIMP1 | PR domain containing 1, with ZNF domain | 0.002045 | class1, cluster 5 | |
| 206121_at | 270 | AMPD1 | adenosine monophosphate deaminase 1 (isoform M) | 0.002656 | no |  |
| 200986_at | 710 | SERPING1 | serpin peptidase inhibitor, clade G (C1 inhibitor), member 1 | 0.002683 | no |  |
| 205983_at | 1800 | DPEP1 | dipeptidase 1 (renal) | 0.002889 | no |  |
| 212419_at | 219654 | ZCCHC24 | zinc finger, CCHC domain containing 24 | 0.003688 | no |  |
| 212868_x_at | 51275 | C12orf47 | chromosome 12 open reading frame 47 | 0.004154 | no |  |
| 215371_at | 9442 | MED27 | similar to cofactor required for Sp1 transcriptional activation, subunit 8, 34kDa; mediator complex subunit 27; CRSP8 pseudogene | 0.004662 | no |  |
| 203027_s_at | 4597 | MVD | mevalonate (diphospho) decarboxylase | 0.004749 | no |  |
| 212086_x_at | 4000 | LMNA | lamin A/C | 0.006221 | no |  |
| 208889_s_at | 9612 | NCOR2 | nuclear receptor co-repressor 2 | 0.008639 | no |  |
