# Supplementary material for: ZFP36L1 Negatively Regulates Plasmacytoid Differentiation of BCL1 Cells by Targeting BLIMP1 mRNA
Source: PLoS One. 2012 Dec 20;7(12):e52187. doi: 10.1371/journal.pone.0052187 (PMC3527407; doi:10.1371/journal.pone.0052187)
Supplement: Table S4 — Enrichment of Gene Ontology terms for ARACNe-inferred ZFP36L1 targets. (DOC) [file pone.0052187.s008.doc]

**Table S4: Enrichment of Gene Ontology terms for ARACNe-inferred ZFP36L1 targets**

| GOID | TERM | CORRECTED_PVALUE | FDR_RATE | ANNOTATED_GENES | |  |  |  |
| --- | --- | --- | --- | --- | --- | --- | --- | --- |
| GO:0032768 | regulation of monooxygenase activity | 0.000838 | 2.00% | GFI1, VDR, APOE |  |  |  |  |
| GO:0051341 | regulation of oxidoreductase activity | 0.00301 | 1.00% | GFI1, VDR, APOE |  |  |  |  |
| GO:0009892 | negative regulation of metabolic process | 0.004392 | 1.33% | GFI1, VDR, NCOR2, BLIMP1, APOE, HMGA1,  SERPING1 | | | | |
| GO:0060558 | regulation of calcidiol 1-monooxygenase activity | 0.005472 | 1.50% | GFI1, VDR |  |  |  |  |
| GO:0009890 | negative regulation of biosynthetic process | 0.007757 | 2.00% | GFI1, VDR, NCOR2, BLIMP1, APOE, HMGA1 | | | |  |
